# Ranking medical & dental institutions based on accreditation standards: Using thermometer to measure atmospheric pressure

**DOI:** 10.12669/pjms.37.7.5229

**Published:** 2021

**Authors:** Saima Chaudhry

**Keywords:** Accreditation, Ranking

***”Rankings depend on what weight we give to what variables”** (Malcolm Gladwell)*^1^.

In Pakistan for 75 years, medical and dental colleges have been ranked on the basis of high school examination and medical college admission test scores, higher the percentage scores required for admission higher the ranking of the institution. This merit has been arbitrarily based on reputation of the alumni of these colleges and the patient load in the attached training hospitals with reference to type and number of patients that the students get exposed to during their training years. However, for first time in history, a formal ranking of the institutions, providing undergraduate medical and dental education is made public by the local accrediting body. The report came out amidst a revamping of the accrediting body itself that has changed from Pakistan Medical & Dental Council (PM&DC) to Pakistan Medical Commission (PMC). In the report, colleges were not numerically ordered but were given academic grades categorising them from A+ (outstanding) to F (Fail). This gained immediate attention of the profession, as some highly prestigious institutions, with a long history of intake of students of highest merit and a large alumni body, providing state of the art services all across the globe, were placed under category ”Fail”.

Probing into the matter it came into light that this categorisation cum ranking was actually based on Accreditation Standards formulated to standardise health professions education across more than 140 public and private sector medical and dental colleges in line with the global guidelines provided by the world federation of medical education (WFME). The then PM&DC decided to sensitise the colleges through an initial inspection and make them aware of the new accreditation criteria to give guidelines for improvement in line with the new standards. This was completed by a thorough inspection and most of the colleges for the first time organised their paperwork to be able to present structured training programs to the accrediting body inspectors. The colleges who had a team that was trained in health professions education performed better at those presentations than those who were doing mostly the right things but unable to present them in the medical education terminologies and jargons. However, all of this initial evaluation exercise was extremely beneficial for guiding the institutions on the need to update medical and dental faculty, infrastructure and curriculum, like incorporation of structured training and quality assessment of graduating doctors.

However, this nevertheless was a program & institution evaluation exercise based on accreditation standards and in this exercise all variables used globally to ”Rank” institutions were not measured.

Identifying this is important, because there is a clear difference between accreditation standards and ranking criteria of institutions both of which are based on different set of performance parameters, viewed from a different lens, thus cannot be used interchangeably. Accreditation is an educational improvement and quality assurance process, whereby a program or institution is assessed to verify whether it continues to meet the norms and standards. Ranking, on the other hand, is an assessment of overall performance of an institution and its comparison with the performance of other institutions.

Accreditation standards are related to vision, mission, infrastructure, faculty, assessment, curriculum indicators and the like, however ranking is generally based on ”beginning characteristics, learning outputs, faculty, learning environment, final outcomes, resources, research, and reputation”^2^. Accreditation ensures minimum standards of performance to ensure professional growth, accountability and public good while ranking is used for institutional branding, to attract best students and faculty in a backdrop of treating education as an industry and professional education as a commodity. The other use of ranking has been the decision for providing funding, the trust of the employer on the quality of the graduates, so takes into account the employability of graduates, and on their market value in terms of salaries. Essentially accreditation is internal to the institution and ranking is external.

Globally methods and indicators used for ranking have attracted much criticism and concern especially where medical and dental institutions are concerned, that are formed with the purpose of service delivery and to provide health as a fundamental right^3^. In which case branding and marketing indicators need to be critically evaluated in line with the vision and mission of the governments and the accrediting bodies. That is why an institutional ranking that fails to present a detailed discussion of methods and variables is dangerous and can misguide the public. Such rankings when issued by government organizations are commonly accepted as authentic and more trusted as compared to the rankings generated by private organisations.

Therefore, to support or refute any ranking, the ranks need to be based on ”reliable and valid data, properly operationalised variables, correctly defined categories, and sound methodologies”^4^. Till that time, when a properly researched methodology is validated in the local context and is made public, the medical and dental colleges cannot be reliably and validly rated. As McGaghie and Thompson state: ”institutions differ in their histories, goals, structures, and aspirations…. in such an environment, a system intended to rate and rank medical schools would need to do so in light of these differences. But even more important, the assessment of quality should be based on criteria of special importance to…. society-that is, the measures should be meaningful in ways that go beyond wealth and reputation”.^5^
